# Efficacy and safety of proton beam therapy for rhabdomyosarcoma: a systematic review and meta-analysis

**DOI:** 10.1186/s13014-023-02223-6

**Published:** 2023-02-20

**Authors:** Meng Dong, Jianrong Wu, Renhua Wu, Dandan Wang, Ruifeng Liu, Hongtao Luo, Yuhang Wang, Junru Chen, Yuhong Ou, Qiuning Zhang, Xiaohu Wang

**Affiliations:** 1 Department of Oncology, Clinical Medical College and Affiliated Hospital of Chengdu University, Chengdu, People’s Republic of China; 2 The First School of Clinical Medicine, Lanzhou University, Lanzhou, People’s Republic of China; 3 Institute of Modern Physics, Chinese Academy of Sciences, No. 1, Yanxia Road, Chenguan District, Lanzhou, 730030 People’s Republic of China; 4 Department of Postgraduate, University of Chinese Academy of Sciences, Beijing, People’s Republic of China; 5 Heavy Ion Therapy Center, Lanzhou Heavy Ions Hospital, Lanzhou, People’s Republic of China

**Keywords:** Particle, Proton therapy, Rhabdomyosarcoma, systematic review, Meta-analysis

## Abstract

**Objective:**

This study aimed to evaluate and conduct a meta-analysis on the efficacy and safety of proton beam therapy (PBT) for rhabdomyosarcoma (RMS).

**Methods:**

We searched for articles using PubMed, Embase, Cochrane Library, and Web of Science databases from their inception to December 22, 2022. Two researchers independently screened literature and extracted data. Statistical analyses were performed using STATA version 14.0.

**Results:**

We got 675 candidate articles, of which 11 studies were included in our study according to the inclusion and exclusion criteria. Of the 544 RMS patients who received PBT. The local control (LC) rate at 1, 2, 3, 4, and 5 years were 96% (95% confidence interval (CI) 0.91–1.01), 93% (95% CI 0.86–1.00), 78% (95% CI 0.71–0.85), 85% (95% CI 0.78–0.92), and 84% (95% CI 0.74–0.95), respectively. The progression-free survival (PFS) rate at 1, 2, 3, 4, and 5 years were 82% (95% CI 0.72–0.92), 73% (95% CI 0.61–0.84), 63% (95% CI 0.47–0.79), 64% (95% CI 0.54–0.74), and 76% (95% CI 0.59–0.94), respectively. The overall survival (OS) rate at 1, 2, 3, 4, and 5 years were 93% (95% CI 0.86–1.00), 85% (95% CI 0.76–0.95), 80% (95% CI 0.63–0.96), 71% (95% CI 0.62–0.80), and 82% (95% CI 0.71–0.94), respectively. Acute and late toxicities were mainly grades 1 to 2 in all studies.

**Conclusion:**

As an advantageous RT technique, PBT is an emerging option for patients with RMS, particularly children and adolescents patients. The data showed that PBT is a feasible, safe, and effective modality for RMS, showing promising LC, OS, PFS, and lower acute and late toxicities.

*PROSPERO registration number*: CRD42022329154.

## Introduction

Rhabdomyosarcoma (RMS) is the most common soft tissue sarcoma in children and adolescents. It is a heterogeneous disease both in presentation and histology, accounting for approximately 5% of all pediatric malignancies [1, 2]. RMS treatment requires multiple modalities, including systemic chemotherapy (CT), local therapy (surgery and radiotherapy), or both. Radiotherapy (RT) is an important treatment strategy for some patients with RMS in unfavorable sites, such as the head, neck, and genitourinary [3–5]. However, radiation oncologists are often very cautious in treating children and adolescents patient with RMS using photon therapy due to long-term toxicity, especially growth retardation and radiation-induced cancer [6–9].

In recent years, advanced radiation modalities have been developed, including proton beam therapy (PBT). They can deposit majority dose in the “Bragg peak” region, providing a more favorable dose-distribution than photons. PBT can deliver a higher dose to the tumor area while protecting the organ at risk from radiation-induced toxicities [10]. As one of the more advanced RT modalities, PBT is a promising treatment strategy for RMS [10].

Clinical studies on PBT for RMS were mainly case series; however, the sample size was small, and the efficacy and safety were not clear and definite. Therefore, this study aimed to systematically evaluate and analyze comprehensive evidence for PBT treatment of RMS and provide the latest evidence for PBT clinical treatment, guideline formulation, and policy implementation.

## Materials and methods

### Literature identification

This systematic review and meta-analysis followed the Preferred Reporting Items for Systematic Reviews and Meta-analysis (PRISMA) guidelines. The review protocol was registered in PROSPERO (CRD42022329154).

### Search strategy

Our search strategy followed the PRISMA guidelines and recommendations [11]. We searched for articles using Cochrane Library, Embase, PubMed, and Web of Science databases from their dates-of-inception to December 22, 2022. Only literature written in English was considered. The search terms were as follows: (“Rhabdomyosarcoma” AND (“Proton therapy OR Proton OR Proton Therap* OR Proton Beam Therap* OR Proton Beam OR Proton Beam Radiation Therapy”)). Simultaneously, the references included in the study were traced to obtain relevant information not found in the above retrieval.

### Inclusion and exclusion criteria

Two researchers (MD and QZ) independently screened all retrieved articles. The inclusion criteria were as follows: (a) studies wherein patients were clinically or pathologically diagnosed with primary or recurrent RMS, and (b) clinical studies reporting incidence of survival outcomes and toxicity in patients who received PBT. In addition, the survival outcome data of these studies were required to identify the overall survival (OS), local control (LC), and progression-free survival (PFS) rates from the initial diagnosis. The exclusion criteria were as follows: (a) studies on patients receiving treatment using only photons, carbon ion RT, brachytherapy, and other particles; (b) duplicate publications; (c) case reports, reviews, meta-analyses, abstracts, letters, comments, and protocols; (d) re-irradiation studies; (e) lack of detailed data; (f) clinical studies with < 10 patients; and (g) other irrelevant topics.

### Data extraction

Literature screening and data extraction of the selected studies were performed by two reviewers (RW and QZ) independently, and the results were checked by a third reviewer (DW). If there was any disagreement, the three investigators discussed it together until a consensus was reached. Data extraction included the following: (a) first author, journal, publication year, country, research institution, study design, and study period; (b) number of patients, age, sex, tumor site, histology, tumor status, stage, intergroup RMS study (IRS) group, risk group, tumor size, total treatment dose, fractions, fraction dose, and follow-up time; (c) the primary outcome was OS, and secondary outcomes were LC, PFS, and toxicity; and (d) evaluation indicators of quality and bias assessments.

### Quality and bias assessments

In our systematic review, each included article was a case series evaluated using the Joanna Briggs Institute (JBI) critical appraisal tool for case series [12]. Literature quality and bias assessments were independently completed by two researchers (QZ and MD). Disputes were resolved by a third reviewer (DW) with answers as yes, no, unclear, or not applicable. The evaluation indicators and outcomes are presented in Table 1.

**Table 1:** Assessment of risk of bias in included studies

References	Criterion									
	a	b	c	d	e	f	g	h	i	j
*USA*										
Ladra et al. [13]	Yes	Yes	Yes	Yes	No	Yes	Yes	Yes	No	Yes
Indelicato et al. [14]	Yes	Yes	Yes	Yes	No	Yes	Yes	Yes	No	Yes
Ludmir et al. [15]	Yes	Yes	Yes	Yes	No	Yes	Yes	Yes	No	Yes
Bradley et al. [16]	Yes	Yes	Yes	Yes	No	Yes	Yes	Yes	No	Yes
Indelicato et al. [17]	Yes	Yes	Yes	Yes	No	Yes	Yes	Yes	No	Yes
Buszek et al. [18]	Yes	Yes	Yes	Yes	No	Yes	Yes	Yes	No	Yes
Parekh et al. [19]	Yes	Yes	Yes	Yes	No	Yes	Yes	Yes	No	Yes
*Japan*										
Mizumoto et al. [20]	Yes	Yes	Yes	Yes	No	Yes	Yes	Yes	No	Yes
Suzuki et al. [21]	Yes	Yes	Yes	Yes	No	Yes	Yes	Yes	No	Yes
*Switzerland*										
Leiser et al. [22]	Yes	Yes	Yes	Yes	No	Yes	Yes	Yes	No	Yes
Weber et al. [23]	Yes	Yes	Yes	Yes	No	Yes	Yes	Yes	No	Yes

(a) Were there clear criteria for inclusion in the case series?; (b) Was the condition measured in a standard, reliable way for all participants included in the case series?; (c) Were valid methods used for identification of the condition for all participants included in the case series?; (d) Did the case series have consecutive inclusion of participants?; (e) Did the case series have complete inclusion of participants?; (f) Was there clear reporting of the demographics of the participants in the study?; (g) Was there clear reporting of clinical information of the participants?; (h) Were the outcomes or follow-up results of cases clearly reported?; (i) Was there clear reporting of the presenting sites’/clinics’ demographic information?; (j) Was statistical analysis appropriate?

### Statistical analysis

Descriptive statistics were used to summarize the baseline variables and incidence of toxicity. Data descriptions included frequencies and percentages for dichotomous data and means with standard deviations or medians with interquartile ranges for continuous data. The case series studies were conducted under different conditions. Thus, we used a random effects model to provide an overall summary estimate. We computed the proportions with 95% confidence intervals (CIs) to estimate the effect sizes for continuous outcomes. All analyses were performed using STATA version 14.0 (StataCorp, College Station, Texas, USA).

## Results

### Study selected and characteristics

**Fig. 1:**
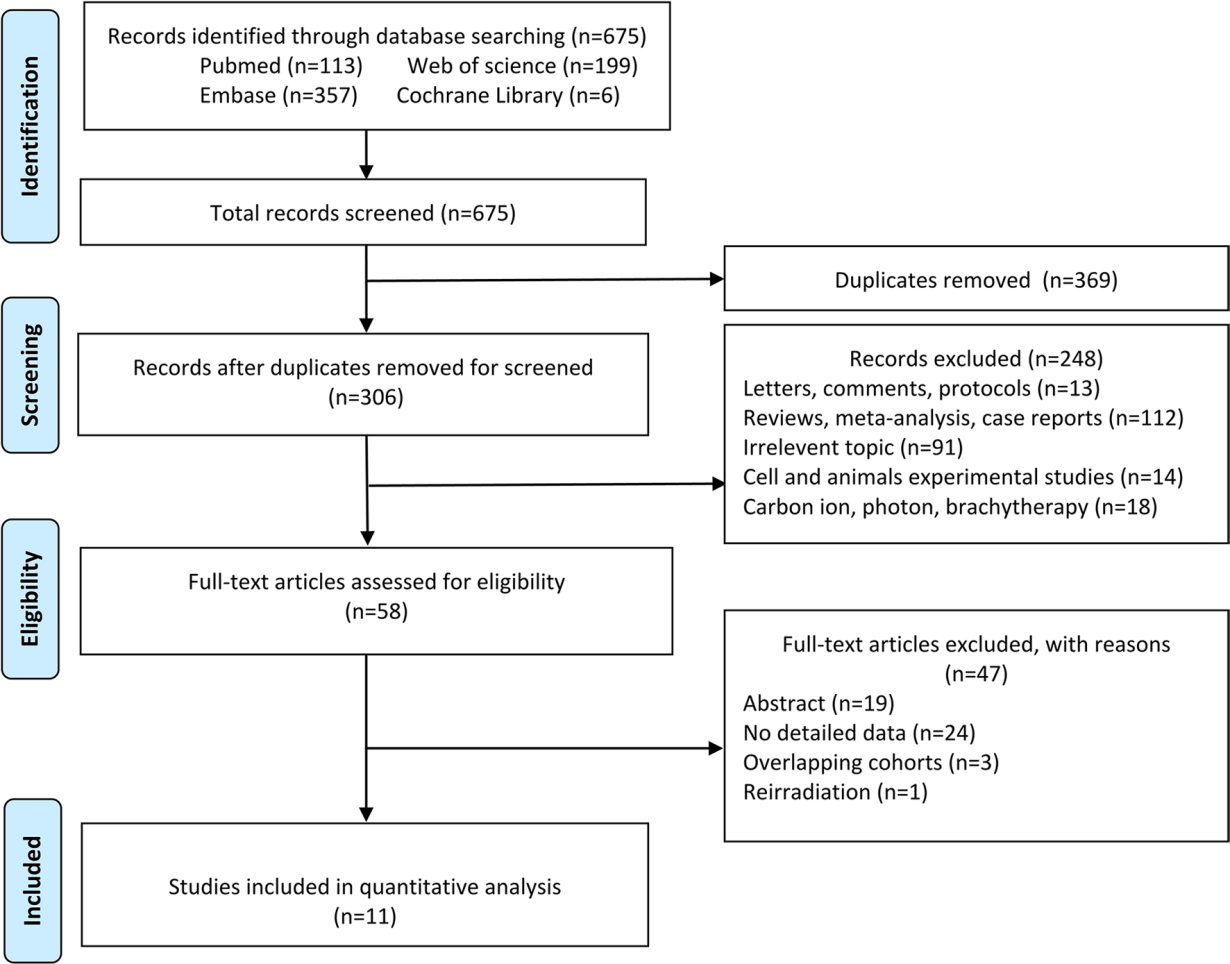
Search results per the PRISMA guidelines

As shown in Fig. 1, the systematic search yielded 675 potential articles for inclusion. After title and abstract reviews, 369 duplicates were removed, resulting in 306 remaining reports. We screened 58 related studies for full-text article eligibility. We eliminated another 47 items, including 19 abstracts, 24 with no detailed data, 3 overlapping cohorts, and 1 re-irradiation, and eventually included 11 articles. These 11 studies originated from 3 countries: the United States (n = 7), Japan (n = 2), and Switzerland (n = 2) [13–23]. The study design included eight prospective and three retrospective studies (Table 2). Only 544 patients with RMS underwent PBT in the studies. These studies reported the survival and toxicity after PBT. Overall, the median sample size was 46 patients (range 24–94), the median age ranged from 15.6 to 69.6 months, the female proportion was 44.1%, and the median follow-up time ranged from 11.52 to 61.2 months (Table 2).

**Table 2:** Baseline characteristics and clinical features of all included studies

References	Study type	No. of patients	Median age (months)	Male/female	Median follow-up (months)	Histology	IRS group	TNM stage	Risk group	Tumor site (favorable*/unfavorable**)	Tumor size (cm)
Ladra et al. [13]	Prospective	57	42 (7.2–235)	27/30	47 (14–102)	Alveolar = 16; Embryonal = 41	Group I = 1; Group II = 7 Group III = 47; Group IV = 2	Stage 1 = 18; Stage 2 = 14 Stage 3 = 23; Stage 4 = 2	Low = 15; High = 0 Intermediate = 42	Favorable = 19; Unfavorable = 38	≤ 5 cm (n = 36); > 5 cm (n = 21)
Indelicato et al. [14]	Prospective	30	57.6 (12–136.8)	19/11	48 (6–114)	Embryonal = 30	Group III = 30	Stage 1 = 30	NR	Favorable = 30; Unfavorable = 0	3.4 cm (range, 2.2–6.1 cm)^£^ 2.3 cm (range, 0.1–4.0 cm)^ǂ^
Ludmir et al. [15]	Prospective	46	64.8 (8.4–188.4)	20/26	46.8 (12–106.8)	Alveolar = 14; Embryonal = 32	Group I = 1; Group II = 5 Group III = 35; Group IV = 5	Stage 1 = 20; Stage 2 = 7 Stage 3 = 14; Stage 4 = 5	Low = 13; High = 5 Intermediate = 28	Favorable = 21; Unfavorable = 25	≤ 5 cm (n = 33); > 5 cm (n = 13)
Bradley et al. [16]	Prospective	24	42 (12–243.6)	12/12	11.52 (3.6–67.2)	Alveolar = 24	Group II = 1; Group III = 23	Stage 2 = 4; Stage 3 = 20	NR	Favorable = 0; Unfavorable = 24	5.5 cm (range, 1.8–9.0 cm)
Indelicato et al. [17]	Prospective	31	31.2 (12–240)	24/7	12 (12–116.4)	Alveolar = 7; Embryonal = 24	Group III = 31	Stage 1/2 = 24 Stage 3 = 7; Stage 4 = 0	NR	Favorable = 0; Unfavorable = 31	≤ 5 cm (n = 6); > 5 cm (n = 25)
Buszek et al. [18]	Prospective	94	39.6 (1.2–187.2)	50/44	48 (4.4–135.6)	Alveolar = 22; Embryonal = 71 Not specified = 1	Group I = 6; Group II = 14 Group III = 62; Group IV = 12	Stage 1 = 25; Stage 2 = 24 Stage 3 = 33; Stage 4 = 12	Low = 19; High = 12 Intermediate = 63	Favorable = 33; Unfavorable = 61	≤ 5 cm (n = 59); > 5 cm (n = 33) Unknown (n = 2)
Parekh et al. [19]	Prospective	37	15.6 (1.2–22.8)	24/13	61.2 (8.4–26.4)	Alveolar = 12; Embryonal = 25	Group I = 1; Group II = 3 Group III = 33; Group IV = 0	Stage 1 = 4; Stage 2 = 5 Stage 3 = 28; Stage 4 = 0	Low = 4; High = 0 Intermediate = 33	Favorable = 9; Unfavorable = 28	≤ 5 cm (n = 13); > 5 cm (n = 24)
Mizumoto et al. [20]	Retrospective	55	60 (0–228)	35/20	24.5 (1.5–320.3)	Alveolar = 18; Embryonal = 31 Others = 6	Group I = 1; Group II = 11 Group III = 37; Group IV = 6	NR	Low = 9; High = 7 Intermediate = 39	Favorable = 37; Unfavorable = 18	NR
Suzuki et al. [21]	Retrospective	48	45.6 (2.4–181.2)	26/22	39.6 (4.8–141.6)	Alveolar = 22; Embryonal = 26	NR	NR	Low = 0; High = 6 Intermediate = 42	Favorable = 25; Unfavorable = 23	NR
Leiser et al. [22]	Prospective	83	54 (9.6–186)	46/37	44 (0.9–126.3)	Alveolar = 9; Embryonal = 74	Group I = 2; Group II = 5 Group III = 65; Group IV = 11	Stage 1 = 22; Stage 2 = 16 Stage 3 = 34; Stage 4 = 11	Low = 20; High = 11 Intermediate = 52	Favorable = 24; Unfavorable = 59	≤ 5 cm (n = 42); > 5 cm (n = 41)
Weber et al. [23]	Retrospective	39	69.6 (14.4–193.2)	21/18	41 (9–106)	Embryonal = 38 Undifferentiated = 1	Group I = 0; Group II = 1 Group III = 33; Group IV = 5	Stage 1/2/3 = 34 Stage 4 = 5	NR	Favorable = 0; Unfavorable = 39	≤ 5 cm (n = 11); > 5 cm(n = 28)

*NR* no reported, *IRS* intergroup rhabdomyosarcoma study group

*Orbital, Head and neck (non-parameningial), Perinea, Biliary, Urogenital (non-bladder/prostate)

**Parameningeal, Bladder/prostate, Extremities, Chest/abdomen, Perianal, Trunk or thorax

^£^The median maximum tumor size at the time of diagnosis

^ǂ^The median maximum tumor size at the time of radiation

### Clinical features

Of the 11 included articles, all patients were diagnosed with RMS. The histology included 392 patients with embryonal, 144 patients with alveolar, and 8 patients with other conditions. Sixty-four percent (n = 346) of the tumors arose in unfavorable sites, and 36 (n = 198) were in favorable sites. The main details of tumor size, stage, risk group, surgery, IRS group, and chemotherapy regimens are shown in Tables 2 and 3.

**Table 3:** Treatment regimens main results of all included studies

References	Surgery n (%)	Chemotherapy n (%)	Beam-delivery	Median total dose (Gy RBE)	Fractions (n)	Dose/fraction Gy_RBE_
Ladra et al. [13]	NR	54 (100%) 18 (31.6%)^c^; 18 (31.5%)^f^; 16 (28.1%)^e^; 3 (5.3%)^h^; 2 (3.5%)^d^	Passive scanning	50.4 (36.0–50.4)	NR	NR
Indelicato et al. [14]	NR	30 (100%)^e/h^	Passive scanning	45	25	1.8
Ludmir et al. [15]	1 (2.2%)	46 (100%) 21 (45.7%)^f^; 10 (21.7%)^e^; 7 (15.2%)^o^; 5 (10.9%)^h^; 3 (6.5%)^g^	Passive scanning Active scanning	50.4 (36.0–50.8)	28	1.8–2.0
Bradley et al. [16]	1 (4.2%)	24 (100%) 15 (62.5%)^h^; 8 (33.3%)^f^; 1 (4.2%)^i^	Passive scanning	50.4 (41.4–59.4)	28	1.8
Indelicato et al. [17]	14 (45.2%)	31 (100%) 19 (61.3%)^a^; 12 (38.7%)^b^	Passive scanning	50.4 (36.0–59.4)	28	1.8
Buszek et al. [18]	60 (63.8%)	94 (100%) 51 (31.6%)^f^; 15 (31.5%)^e^; 10 (28.1%)^g^; 9 (5.3%)^a^; 6 (3.5%)^0^; 3 (3.5%)^c^	Passive scanning Active scanning	50.4 (36.0–50.8)	28	1.8–2.0
Parekh et al. [19]	20 (54.1%)	37 (100%) 18 (48.7%)^a^; 17 (45.9%)^b^; 2 (5.4%)^o^	Passive scanning	50.4 (36.0–55.8)	28	1.8
Mizumoto et al. [20]	41 (74.5%)	53 (96.4%)^NR^	NR	50.4 (36.0–60.0)	NR	NR
Suzuki et al. [21]	21 (43.8%)	46 (95.8%) 40 (83.3%)^j^; 6 (12.5%)^k^	NR	50.4 (41.4–59.4)	NR	NR
Leiser et al. [22]	55 (66.3%)	83 (100%) 59 (71.1%)^l^; 14 (16.9%)^a^; 5 (6.0%)^m^; 3 (3.6%)^f^; 1 (1.2%)^c^; 1 (1.2%)^n^	Active scanning	54 (41.4–64.8)	30	1.8–2.0
Weber et al. [23]	NR	39 (100%) 28 (71.8%)^l^; 5 (12.8%)^a^; 3 (7.7%)^b^; 3 (7.7%)^m^	Active scanning	54 (50.4–55.8)	30	1.8–2.0

*EpSSG* European Pediatric Soft Tissue Sarcoma Study Group, *COG* Children’s Oncology Group, *NR* no reported, *RMS* Rhabdomyosarcoma, *CWS* Cooperative Weichteilsarkom Studies, *MSKCC* Memorial Sloan Kettering Cancer Center, *RBE* Relative Biologic Effectiveness

^a^EpSSG regimens; ^b^: COG regimens; ^c^: COG-D9803; ^d^: D9602; ^e^: COG-ARST0331; ^f^: COG-ARST0531; ^g^: COG-ARST0431; ^h^: EpSSG 2005; ^i^: St Jude RMS 13; ^j^: VC (vincristine and cyclophosphamide); ^k^: Irinotecan-containing regimen; ^l^: CWS (2002P/ Guidance 2006/2007 HR/ Guidance 2009/ DOK IV 2004); ^m^: SIOP-MMT-95; ^n^: MSKCC 03,099; ^o^: other regimens

### Proton beam therapy

In terms of PBT, each research center used a different beam delivery system (Table 3). Passive scanning is mainly performed in the United States, whereas active scanning is mainly performed in Switzerland. Regarding the total dose, each research center used different dose fractions (Table 3).

### LC, PFS, and OS rate outcomes of PBT

**Fig. 2:**
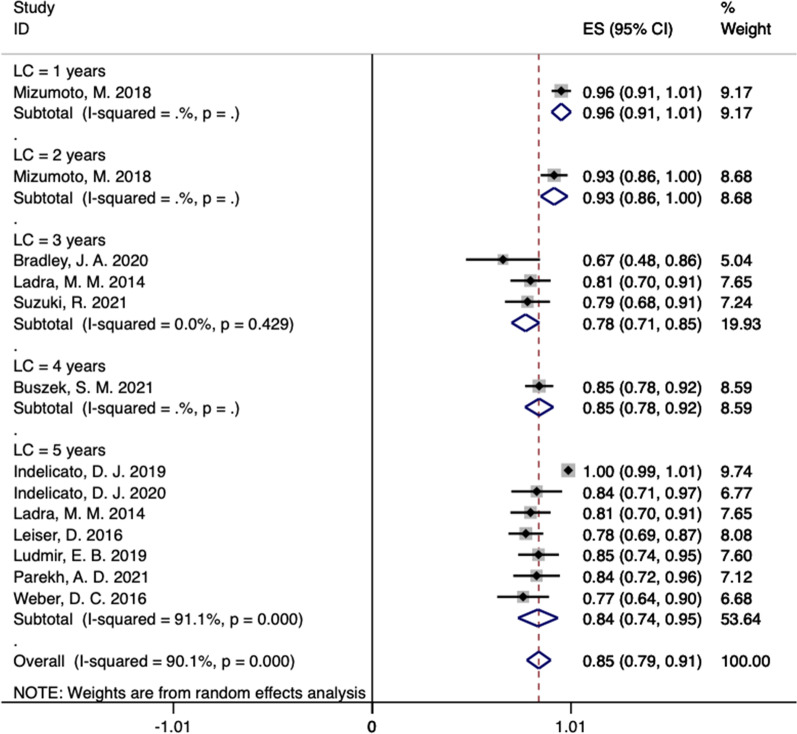
The pooled incidences of LC after PBT for RMS

**Fig. 3:**
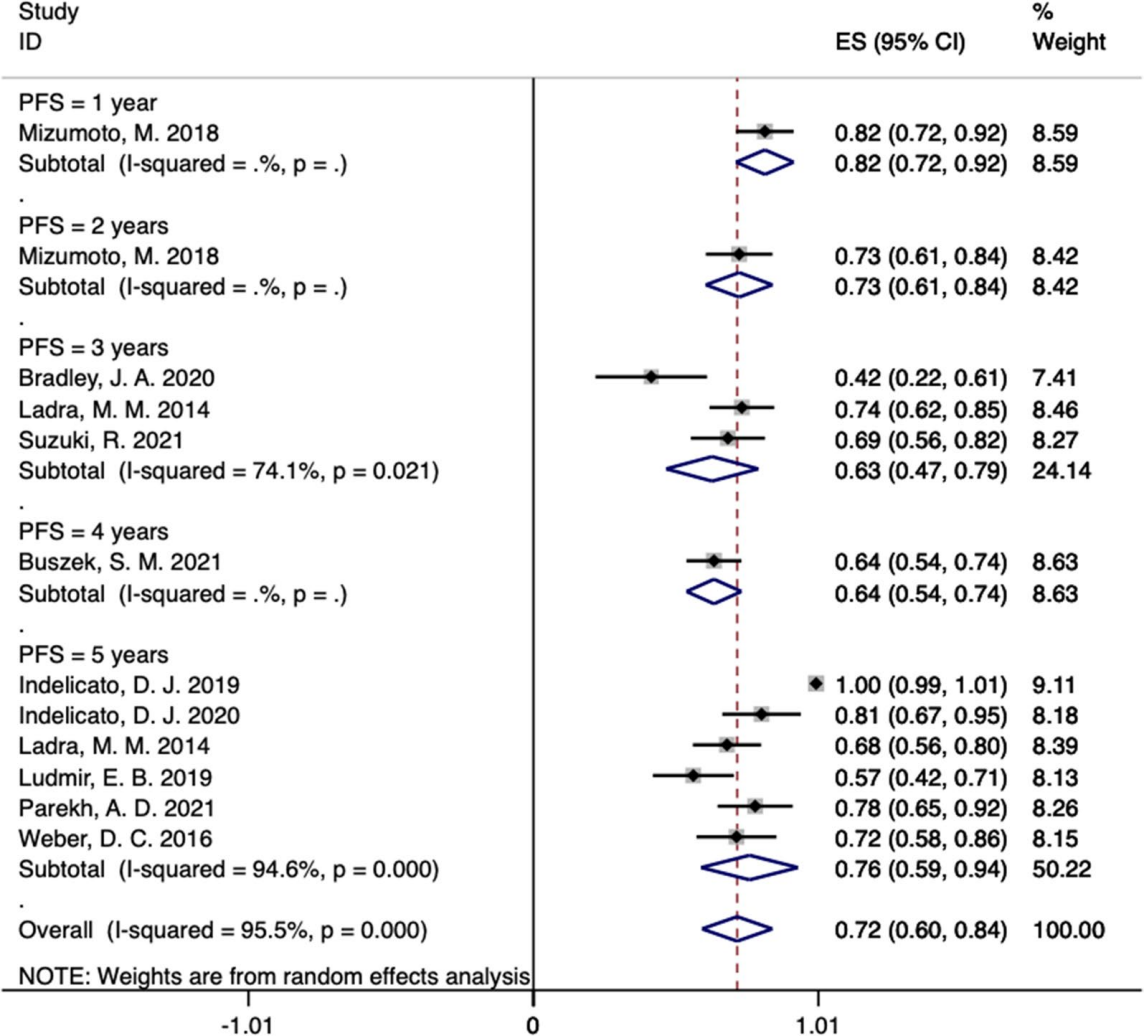
The pooled incidences of PFS after PBT for RMS

**Fig. 4:**
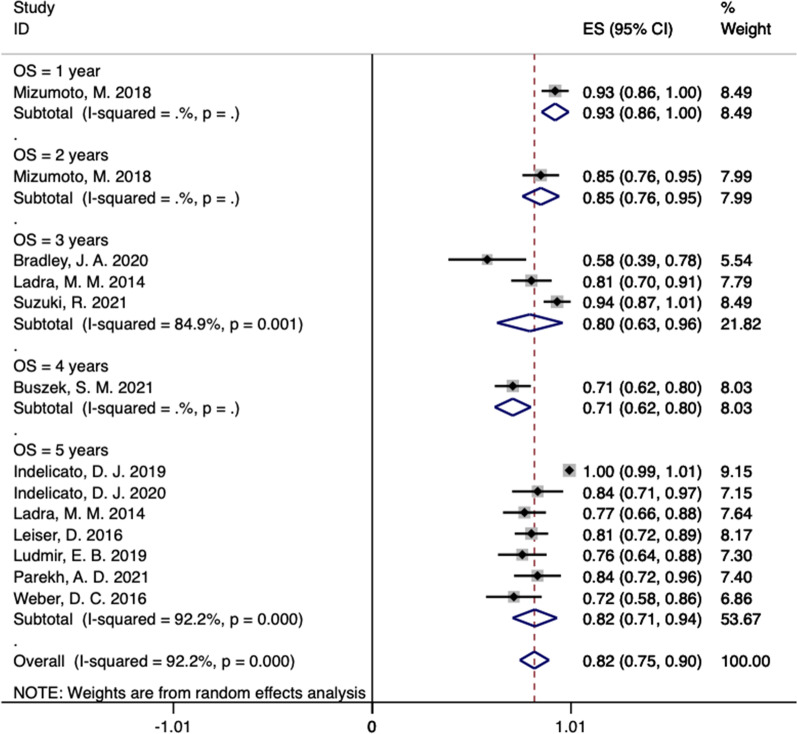
The pooled incidences of OS after PBT for RMS

In our systematic review, the LC incidence at 1, 2, 3, 4, and 5 years in these studies were 96% (95% CI 0.91–1.01), 93% (95% CI 0.86–1.00), 78% (95% CI 0.71–0.85, *I*^2^ = 0%), 85% (95% CI 0.78–0.92), and 84% (95% CI 0.74–0.95, *I*^2^ = 91.1%), respectively (Fig. 2) [13–23]. In ten studies reported the PFS rate outcomes of PBT for RMS (Fig. 3) [13–21, 23]. The PFS rate at 1, 2, 3, 4, and 5 years in these studies were 82% (95% CI 0.72–0.92), 73% (95% CI 0.61–0.84), 63% (95% CI 0.47–0.79, *I*^2^ = 74.1%), 64% (95% CI 0.54–0.74), and 76% (95% CI 0.59–0.94, *I*^2^ = 94.6%), respectively (Fig. 3) [13–21, 23]. As shown in Fig. 4, after undergoing PBT for 1, 2, 3, 4, and 5 years, the OS rates for RMS were 93% (95% CI 0.86–1.00), 85% (95% CI 0.76–0.95), 80% (95% CI 0.63–0.96, *I*^2^ = 84.9%), 71% (95% CI 0.62–0.80), and 82% (95% CI 0.71–0.94, *I*^2^ = 92.2%), respectively [13–23].

### Toxicity

Across all studies, the incidence of acute and late toxicities were mainly grades 1 to 2 (Table 4). Acute toxicity grade 3 was observed in 6 studies. The incidence of which was 9–25% [13, 15, 16, 20–22]. Late toxicity grade 3 was observed in 7 articles, with an incidence of 2.1–26% [13, 15–17, 19, 22, 23]. Acute toxicity of orbital embryonal RMS were mild. Serious late toxicities included cataracts (n = 18), permanently reduced visual acuity (n = 4), and chronic sinusitis (n = 1) [14]. Two studies of parameningial RMS (PM-RMS) identified grade 3 late toxicity of unilateral cataracts and hearing impairment; however, the incidence was 8% [16, 23]. The study of head and neck RMS (H&N-RMS) identified grade 3 acute and late toxicity; the incidence were 9%, and 26%, respectively [15]. Regarding RMS in the pelvis, we observed grade 2 acute toxicity and grade 3 late toxicity, the incidence of which were 16% and 6%, respectively [17]. Dysfunction was reported in three studies, including unilateral hearing loss, cognitive disturbance, and skeletal muscle defect; however, grade 3 dysfunction occurred in only one case [13, 15, 20]. In addition, two studies reported secondary malignancy (radiation-induced); the incidence of which were 1.8% (n = 1) and 2.4% (n = 2), respectively [20, 22].

**Table 4:** Survival outcomes, toxicity incidence and prognostic factors on patients of all included studies

References	Local failure n (%)	Regional failure n (%)	Metastasis n (%)	Radiation induced cancer n (%)	Local control	Progress-free survival	Overall survival	Toxicity	Prognostic factors
Ladra et al. (2014) [13]	10 (18%)	4 (7%)	5 (9%)	0	3-y (81%) 5-y (81%)	3-y (73%) 5-y (69%)	3-y (81%) 5-y (78%)	Acute: ≤ G3 (G3 = 23%) Late: ≤ G3 (G3 = 5%)	Age (˂ 2 y vs 2–10 y vs > 10 y); Tumor size (> 5 cm vs ≤ 5 cm); **Stage (1 to 2 vs 3 to 4) **^**b**^; **Risk group (Low vs Intermediate) **^**b**^; Histology (Embryonal/botryoid vs Alveolar/undifferentiated); Group (I to II vs III to IV); Site (Favorable vs Unfavorable)
Indelicato et al. [14]	1 (3%)	0	0	0	5-y (97%)	5-y (97%)	5-y (100%)	Acute: Mild Late: Unable to evaluate	NR
Ludmir et al. [15]	7 (15%)	9 (20%)	4 (9%)	0	5-y (84%)	5-y (57%)	5-y (76%)	Acute: ≤ G3 (G3 = 9%) Late: ≤ G3 (G3 = 26%)	**Primary tumor size (> 5 cm vs ≤ 5 cm)**^**d**^; **The presence of intracranial extension at diagnosis**^**d**^; Primary tumor site; Age; Histology; Study protocol; Post-surgical IRS risk group; Cyclophosphamide equivalent dose; PBS-PT vs PSPT; Radiotherapy dose; Radiotherapy timing
Bradley et al. [16]	6 (25%)	0	7 (29%)	0	3-y (66%)	3-y (40%)	3-y (58%)	Acute: ≤ G3 Late: ≤ G3	**Age (˂ 4 y vs ≥ 4 y)**^**e**^; Weeks from chemotherapy to radiation therapy (˂ 14 weeks vs ≥ 14 weeks); Elapsed days (˂ 39 vs ≥ 39); **Intracranial extension **^**b,e**^; **Nodal stage (0 vs 1)**^**b,e,f**^; **Primary tumor size (˂ 5 cm vs ≥ 5 cm)**^**e**^; Race (White vs Other); **Sex**^**a**^; Total dose (˂ 50.4 GyRBE vs ≥ 50.4 GyRBE)
Indelicato et al. [17]	4 (13%)	0	2 (6%)	0	5-y (83%)	5-y (80%)	5-y (84%)	Acute: ≤ G2 (G2 = 16%) Late: ≤ G3 (G3 = 6%)	**Age (0–2 y vs ≥ 3 y)**^**a**^; Sex; Ethnicity (White vs Other); **Histology (Alveolar vs Embryonal)**^**b**^; Regional lymph nodes (Positive vs Negative); Chemotherapy regimen (EpSSG vs COG); Median duration between chemotherapy and start of radiation (˂ Median vs ≥ Median); Total dose (> 50.4GyRBE vs ≤ 50.4GyRBE); Maximum size at diagnosis (˂ 5 cm vs 5-8 cm vs > 8 cm); Volume at diagnosis (˂ 92cm^3^ vs 93-382cm^3^ vs > 383cm^3^); Surgery (Resected vs Unresected)
Buszek et al. [18]	12 (13%)	20 (21%)	7 (7%)	0	4-y (85%)	4-y (63%)	4-y (71%)	Acute: NR Late: NR	**Tumor size (≤ 5 cm vs > 5 cm)**^**a,b,c**^**; Timing of radiotherapy to chemotherapy (≤ 13 weeks vs > 13 weeks)**^**a,b,c**^; Cyclophosphamide dose; **Intermediate-risk rhabdomyosarcoma**^**d**^
Parekh et al. [19]	8 (22%)	0	1 (3%)	0	5-y (83%)	5-y (78%)	5-y (83%)	Acute: No Late: ≤ G3 (G3 = 16%)	Age (12 months vs > 12 months); Gender; **Site (Favorable vs Unfavorable)**^**a,b,c**^**; Histology (Alveolar vs Embryonal)**^**a,b**^; Size (5 cm vs > 5 cm); **Stage (I/II vs III)**^**b,c**^; Nodal disease (N0 vs N1); Chemotherapy (COG vs Other); Timing of radiotherapy to chemotherapy (≤ 12 weeks vs > 12 weeks)
Mizumoto et al. [20]	5 (9%)	0	8 (15%)	1 (1.8%)	1-y (96%) 2-y (93%)	1-y (82%) 2-y (72%)	1-y (92%) 2-y (85%)	Acute: ≤ G3 (G3 = 16%)^ǂ^ Late: ≤ G2 (G2 = 15%)	**COG Risk group**^**a,b**^
Suzuki et al. [21]	9 (19%)	0	3 (6%)	0	3-y (79%)	3-y (69%)	3-y (94%)	Acute: ≤ G3 (G3 = 25%) Late: Unable to evaluate	NR
Leiser et al. [22]	20 (24%)	0	4 (5%)	2 (2.4%)	5-y (79%)	NR	5-y (81%)	Acute: ≤ G3 (G3 = 15%) Late: ≤ G3 (G3 = 18%)	Age at first diagnosis (≥ 4.5y); Age at first diagnosis (≤ 10y); Gender; **Tumour site (Other vs PM)**^**a**^; **IRS Group (≥ IIIb)**^**a**^; **COG Stage (≥ 3)**^**a**^; **COG Risk group (High vs low/int.)**^**a**^; Histology of disease (Alveolar vs Embryonal); **Size at diagnosis (> 5 cm)**^**a**^; Positive lymph node at diagnosis; Total dose (≥ 54 GyRBE); **In PM RMS (Intracranial extension)**^**a**^
Weber et al. [23]	9 (23%)	0	2 (5%)	0	5-y (77%)	5-y (72%)	5-y (73%)	Acute: NR Late: ≤ G3 (G3 = 8%)	**Interval time (IT) between the start of the neoadjuvant chemotherapy and start of the proton therapy (> 13 weeks) **^**c**^

Boldface indicates statistically significant difference

*NR* no reported, *IRS* Intergroup Rhabdomyosarcoma Study, *EpSSG* European Pediatric Soft Tissue Sarcoma Study Group, *COG* Children’s Oncology Group, *PM* Parameningial, *RMS* Rhabdomyosarcoma, *RBE* Relative Biologic Effectiveness, *PBS-PT* Pencil-beam Scanning Proton Beam Therapy, *PSPT* Passivescattered Proton Beam Therapy

ǂRadiation- induced toxicities (including mucositis and dermatitis)

^a^Factor significantly correlated with local control (LC) (p ≤ 0.05); ^b^factor significantly correlated with overall survival (OS) (p ≤ 0.05); ^c^factor significantly correlated with progress-free survival (PFS) (p ≤ 0.05); ^d^factor significantly correlated with local failure (LF) (p ≤ 0.05); ^e^factor significantly correlated with freedom from metastases (p ≤ 0.05); ^f^factor significantly correlated with disease-free survival (p ≤ 0.05)

### Prognostic factors of PBT effectiveness

In our systematic review, nine studies reported the prognostic factors of PBT effectiveness. The following factors were evaluated: age, sex, race, tumor size, surgery, risk group, histology, IRS group, lymph nodal stage, intracranial extension, beam delivery system, interval time between RT and CT, CT regimens or dose, and total dose. Table 4 (Boldface indicates statistically significant difference) shows the main details of the prognostic factors of PBT effectiveness in all the included studies [13, 15–20, 22, 23].

## Discussion

In the past 40 years, significant advances have been made in treating RMS, with a 3-year OS rate of approximately 80% in high-risk patients [24]. RT is an important part of the current pretreatment strategy for RMS, and the absence of RT for high-risk RMS leads to a poor prognosis. According to previous studies, PBT was used to treat various malignancies. The supposed decrease of toxicity and better therapeutic effect with proton therapy are not strictly confirmed but strongly supported according to dosimetric comparisons [25]. We analyzed all available studies on PBT for treating RMS, including efficacy, safety, and prognostic factors. Our study found that PBT is safe and effective for RMS, showing promising results for LC, PFS, and OS and acceptable acute and late toxicities.

In our systematic review, patients with RMS were treated using PBT. The median total dose was 45–50.4 Gy_RBE_; most research institutions used a median total dose of 50.4 Gy_RBE_ (Table 3). In terms of included patients, pooled probabilities of LC, PFS, and OS were 85%, 72%, and 82%, respectively (Figs. 2, 3, 4). According to previous clinical outcomes based on photon RT, LC rates were 62–88%. Therefore, compared with previous clinical reports, the efficacy of PBT for RMS is comparable to that of photon RT [5, 24, 26–28].

PM-RMS is a mesenchymal tumor that usually invades the leptomeninges into the brain, leading to neoplastic meningitis [29]. PM-RMS accounts for approximately 40% of H&N tumors and 15% of all RMS in children and is a refractory RMS [3, 30]. In two studies on PM-RMS with PBT in our review (Table 4), the 5 years LC, PFS, and OS were 77%, 72%, and 73%, respectively [23]. In terms of X-ray RT for PM-RMS, Merks et al. reported a study of 862 patients who received RT [31]. The event-free survival (EFS) rates at 5 and 10 years for all patients were 64.9% and 62.6%, respectively; the OS rates at 5 and 10 years for all patients were 69.5% and 66.1%, respectively. In addition, their study also showed that patients with PM-RMS who did not receive RT had worse OS rates (5 years OS 49.6% versus 71.4%; 10 years OS 40.8% versus 68.5%) [31]. These studies showed that proton and photon therapies have similar clinical outcomes for both disease control and survival.

In patients with RMS, unfavorable tumor sites often predict poorer disease control and survival [19, 22]. These usually include parameninges, bladder/prostate, extremities, chest/abdomen, perianal, and trunk or thorax. In contrast, favorable tumor sites include the orbital, head, neck (non-parameningeal), perineal, biliary, and urogenital (non-bladder/prostate). We included three studies with unfavorable disease sites (Table 2) [16, 17, 23]. The LC, PFS and OS incidence at 3 years in these studies were 66%, 40%, and 58%, respectively; the LC, PFS and OS incidence at 5 years in these studies were 77–83%, 72–80%, and 73–84%, respectively (Table 4) [16, 17, 23]. These results suggest that, despite irradiating unfavorable sites of RMS with a higher median total dose of PBT (50.4–54 Gy_RBE_), local failure is expected to occur within 3 or 5 years in 13–25% of the cases [16, 17, 23]. Regarding favorable sites of RMS, Indelicato et al. reported orbital RMS treated with PBT. The LC, PFS, and OS rates at 5 years were 97%, 97%, and 100%, respectively [14]. The results of this study suggest that 45 GyRBE PBT for favorable RMS sites maybe achieve satisfactory disease control and survival.

Balancing disease control with toxicity remains a significant challenge for radiation oncologists since it is the most common soft tissue sarcoma in children and adolescents. In our systematic review, the incidence of acute and late toxicities was mainly grade 1 to grade 2 (Table 4). The most common event was an acute skin or mucous membrane reaction [13–15, 20–22], with an incidence of grade 3 acute toxicity at 4–9% and 2–12%, respectively [13, 15, 20, 22]. No grade 4 or higher skin and mucosal acute reactions were observed in any of the studies. Gaito et al. reported radiation-induced skin toxicity (RIST) profile of photon radiotherapy versus PBT in patients with RMS and Ewing sarcoma [32]. With regards to acute RIST, 47.9% of photon radiotherapy patients and 48.4% of PBT patients had acute grade 2/3 toxicity. When it comes to late RIST, 17.5% of photon radiotherapy patients and 29.0% of PBT patients had grade 1/2 toxicity. This difference in grade 1/2 toxicity between photon radiotherapy and PBT was not statistically significant (*P* = 0.25) [32]. In terms of late toxicity, grade 3 was observed in 7 articles, with an incidence of 5–26% [13, 15–17, 19–23]. RMS of the H&N did not present with more than grade 3 early and late toxicities [14–16, 23]. One study of pelvic RMS had grade 3 late toxicity, including gonadal failure, stress fracture of S1, and leg length discrepancy; however, the incidence was 6% [17]. Parekh et al. reported 37 cases of infant RMS (˂ 24 months) without acute toxicity; however, grade 3 late toxicity was observed in 6 patients, including cataract (11%), eyelid entropion (3%), and scoliosis (3%) [19]. Dysfunction was reported in three studies, including unilateral hearing loss, cognitive disturbance, and skeletal muscle defect; however, only one case of grade 3 dysfunction was observed [13, 15, 20]. Additionally, two studies reported secondary malignancy (radiation-induced); the incidence were 1.8% (n = 1) and 2.4% (n = 2), respectively [20, 22]. Although the toxicity of PBT was low and acceptable, late toxicity, especially dysfunction and secondary malignancy (radiation-induced) required larger samples and long-term follow-up.

In our systematic review, nine studies reported the prognostic factors of PBT effectiveness (Table 4) [13, 15–20, 22, 23]. The following factors were evaluated: age, sex, race, tumor size, surgery, risk profile, histology, IRS group, lymph nodal stage, intracranial extension, beam-delivery system, interval time between RT and CT, CT regimens or dose, and total dose. Prognostic factors varied widely among the selected studies. Overall, most studies showed that risk group, tumor size, tumor site, stage, and intracranial extension are common significant prognostic factors for RMS. Furthermore, younger age, shorter interval time between RT and CT, and negative lymph nodal stage were significantly associated with better LC, PFS, and OS. According to Kubo et al., the PAX3/7-FOXO1 fusion gene may be a potential unfavorable prognostic factor [33]. There were three articles reporting about the PAX3/7-FOXO1 fusion gene in our study, but no correlation was found with survival prognosis [15, 16, 19].

This systematic review and meta-analysis had several limitations. First, grey literature were not included, and there may be publication bias. Second, our search results showed that 64% of the literature on PBT for RMS was from the United States, 18% was from Japan, and 18% was from Switzerland. Therefore, reporting bias may be present. In addition, all studies were case series reports without randomized controlled studies and included small sample sizes. This may affect the reliability of the conclusions of this systematic review. Third, due to limited data, conducting a subgroup analysis of disease control and survival for different histology, IRS group, stage, and risk group was difficult. However, all study designs were reasonable, the missed follow-up rates were low, and the strength of the endpoints was high, with all studies evaluating LC, PFS, and OS as specific outcomes.

As an advantageous RT technique, PBT has shown promising efficacy and acceptable toxicity in RMS treatment. However, there are still some areas of insufficient PBT for RMS. First, previous studies on PBT for RMS often involved different age groups, sites, IRS groups, risk groups, and stages. Different types of RMS may have inconsistent optimal dose patterns, and individualized PBT requires further study. Second, although PBT for RMS has achieved good LC and PFS, integrated treatment modalities, including CT regimens, anti-angiogenic therapy, and immunotherapy, require further study. Third, the number of patients treated with PBT for RMS was too small, although a potential role of protons in improving LC and PFS at low toxicity was found. In addition, the relatively short follow-up period of the current study limits the reliability of the long-term toxicity evaluation of proton therapy for RMS, such as recurrence, functional deficits, growth and development, and secondary cancer. Lastly, whether PBT is superior to other RT technologies needs to be determined with high-quality prospective randomized controlled clinical trials in patients with RMS.

## Conclusion

As an advantageous RT technique, PBT is an emerging option for patients with RMS, particularly children and adolescents patients. The data showed that PBT is a feasible, safe, and effective modality for RMS, showing promising LC, OS, PFS, and lower acute and late toxicities. However, whether PBT is superior to other RT technologies needs to be determined using high-quality prospective randomized controlled clinical trials.

## Data Availability

All data are provided.

## Abbreviations

PBT: Proton beam therapy
RMS: Rhabdomyosarcoma
LC: Local control
CI: Confidence interval
PFS: Progress-free survival
OS: Overall survival
CT: Chemotherapy
RT: Radiotherapy
H&N-RMS: Head and neck rhabdomyosarcoma
PRISMA: Preferred Reporting Items for Systematic Reviews and Meta-analysis
IRS: Intergroup rhabdomyosarcoma study
RBE: Relative biological effectiveness
JBI: Joanna Briggs Institute
CI(s): Confidence interval(s)
PM-RMS: Parameningial rhabdomyosarcoma
